# Monocarboxylate Transporter 1 (MCT1) in Cancer Biology: Canonical Transport Functions, Metabolic–Epigenetic Crosstalk and Emerging Nuclear Localisation

**DOI:** 10.3390/cancers18162699

**Published:** 2026-08-20

**Authors:** Jakub Franczak, Ayşe Latif

**Affiliations:** Experimental Oncology Group, Stopford Building, Division of Pharmacy & Optometry, Faculty of Biology, Medicine and Health, The University of Manchester, Oxford Road, Manchester M13 9PL, UK; jakub.franczak@manchester.ac.uk

**Keywords:** monocarboxylate transporter 1, MCT1, *SLC16A1*, cancer metabolism, lactate transport, metabolic reprogramming, nuclear localisation, epigenetic regulation, tumour microenvironment, MCT1 inhibitors

## Abstract

MCT1 (encoded by *SLC16A1*) is a key regulator of cellular metabolism, mediating proton-coupled transport of lactate, pyruvate, ketone bodies, and other monocarboxylates across biological membranes. Long recognised for its canonical role in metabolic homeostasis and the lactate shuttle, MCT1 is now implicated in tumour-promoting processes, including metabolic symbiosis, angiogenesis, immune evasion, and therapy resistance. Aberrant plasma membrane MCT1 (PM MCT1) expression is observed in diverse malignancies, where it may carry prognostic or predictive value, making it an attractive therapeutic target. This review integrates established metabolic functions of MCT1 with emerging evidence showing its unexpected nuclear localisation (nMCT1) and potential to modulate chromatin state through metabolite-driven epigenetic regulation. In particular, we discuss how PM MCT1 substrates such as lactate, pyruvate, and ketone bodies may influence histone modifications and gene regulation through direct or indirect metabolic mechanisms. We also examine reports of nuclear or nuclear-associated MCT1 (nMCT1) staining in immune and cancer contexts, while emphasising that functions of nMCT1 remain insufficiently validated. By distinguishing established transport biology from substrate-mediated epigenetic effects, this review highlights both the therapeutic promise of MCT1 targeting and the experimental gaps that must be addressed. We conclude by outlining priorities for future research, including orthogonal validation of putative nMCT1, improved patient stratification based on MCT1 expression and metabolic phenotype, and rational combination strategies for MCT1-directed therapies.

## Simple Summary

Cancer cells often change how they use nutrients to grow, survive, and resist treatment. MCT1 is found at increased levels in many cancers, where it supports the transport of small molecules such as lactate and pyruvate in and out of cells. This allows cancer cells to change their metabolism and survive in nutrient-deprived environments. Although this protein is best known for its role in metabolism at the plasma membrane, recent evidence suggests that it may also localise to the nucleus, the command centre of the cell, where it may have unexpected functions. This review brings together current knowledge on how MCT1 contributes to cancer progression, treatment resistance, and changes in gene activity. By highlighting both established and emerging roles, this review aims to clarify why this transporter is clinically important and how it could be targeted more effectively in future cancer therapies.

## 1. Introduction

Cancer biology is undergoing a paradigm shift. Once viewed primarily through the lens of genetic mutations and signalling pathway dysregulation, it is now increasingly understood as a disease driven by dynamic interactions between metabolism, epigenetics, and the tumour microenvironment. Membrane transporters in the solute carrier (SLC) superfamily are central to this integration, regulating metabolite movement across membranes to support rapid adaptation to nutrient availability, environmental stress, and proliferative demands.

Within this superfamily, the *SLC16A* family includes monocarboxylate transporters involved in the movement of monocarboxylic acids such as lactate, pyruvate, butyrate and ketone bodies. In mammalian cells, these metabolites cross lipid bilayers through MCTs, sodium-coupled monocarboxylate transporters, or mitochondrial pyruvate carriers [1,2,3]. Of the fourteen *SLC16A* family members, four proteins (MCT1–4) mediate proton-coupled transport of monocarboxylic acids across the lipid bilayer, while the remainder are hypothesised to function in a H^+^-independent manner [4]. MCT1 was the first transporter to be molecularly characterised via cDNA cloning [5]. Early work using *Xenopus laevis* oocytes revealed that MCT1 functions as an intermediate-affinity transporter for monocarboxylates capable of both import and export. It can also operate as a proton-independent exchanger, swapping one monocarboxylate for another [6,7].

**Figure 1:**
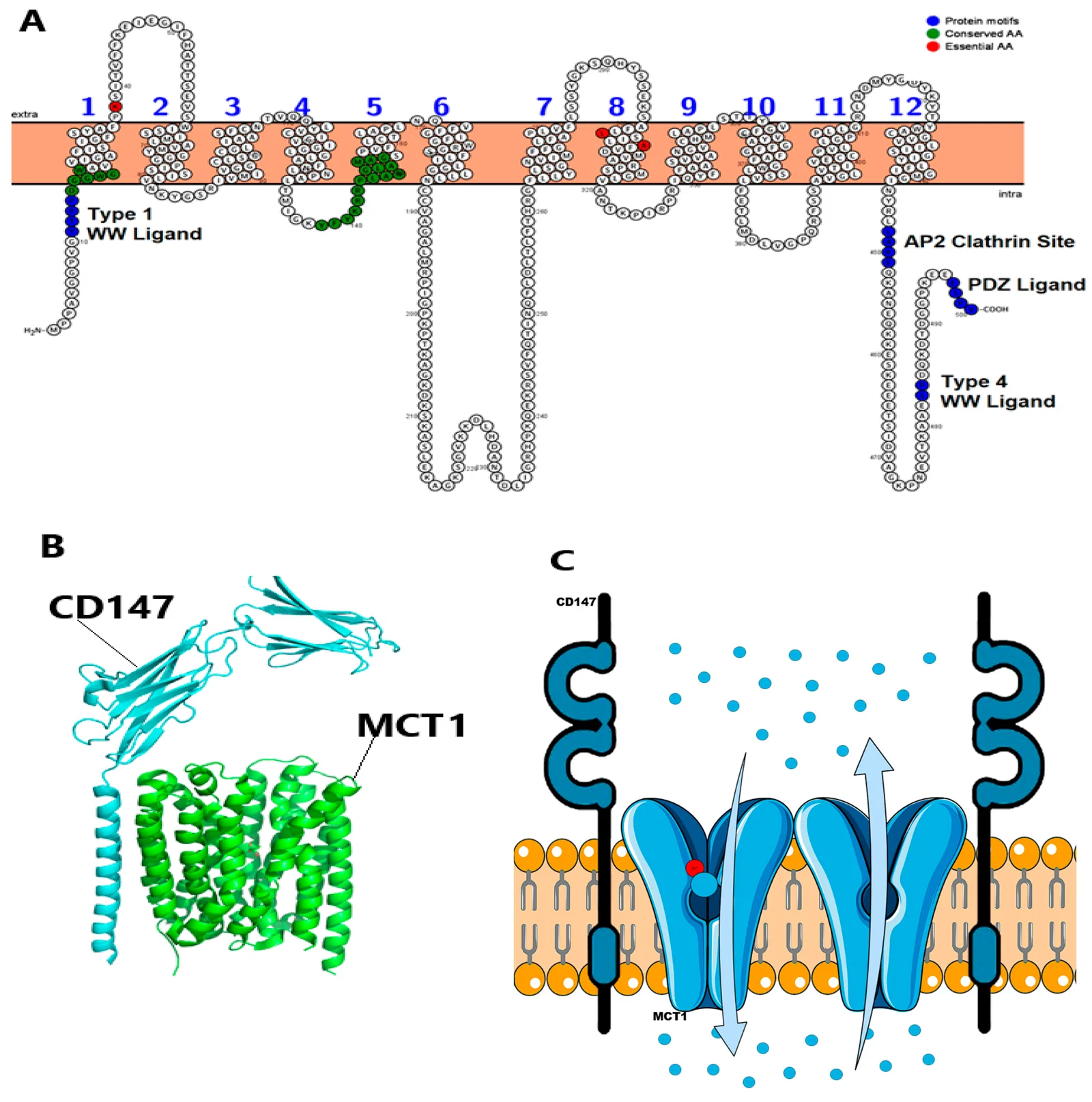
Structure and function of Monocarboxylate Transporter 1 [10,27,28]. This figure illustrates the membrane topology and structure of Monocarboxylate Transporter 1 (MCT1). (**A**) The topology of MCT1 contains twelve transmembrane domains, assembled into N- and C-terminal bundles of six. Bundle rearrangement drives proton-coupled transport of monocarboxylates across the lipid bilayer. Essential amino acids required for transporter activity are highlighted in red. Highly conserved peptide sequences found amongst other MCT family members are highlighted in green. Protein motifs found in N- and C-termini of MCT1, as identified by Uhernik et al., are highlighted. Created using Protter software. In panel A, numbers 1–12 indicate the twelve predicted transmembrane domains of MCT1; red highlights essential amino acids, green highlights conserved amino acids/sequences, and blue highlights annotated protein motifs. (**B**) CryoEM structure MCT1 (green) in the outward open conformation associated with its preferred chaperone CD147 (blue), PDB ID: 6LZ0. (**C**) The functional MCT1 complex is hypothesised to consist of MCT1 homodimer bound to two CD147 molecules. The transporter complex facilitates the bidirectional transport of monocarboxylate acids across the plasma membrane. In panel C, the arrows indicate bidirectional monocarboxylate transport across the plasma membrane.

Structurally, MCT1 contains twelve transmembrane α-helices arranged in N- and C-terminal bundles of six, connected by a 30 amino acid cytoplasmic loop (Figure 1) [8]. Studies using site-directed mutagenesis, chemical labelling, and cryo-EM have revealed both “open” and “closed” conformations, supporting a rocker-switch transport mechanism [9,10]. Unlike many SLC transporters, MCT1 does not rely on N-glycosylation or other post-translational modifications for membrane trafficking [11]. Instead, it requires association with immunoglobulin superfamily chaperones, primarily CD147 (basigin) or gp70 (embigin) [12,13]. MCT1 and its chaperones show co-dependence, and experimental evidence demonstrates that failure to associate with CD147 results in the retention of both proteins in the endoplasmic reticulum and the Golgi apparatus [13]. Fluorescence resonance energy transfer (FRET) studies demonstrated that functional MCT1 complex forms a heterotetramer, consisting of an MCT1 homodimer bound to two CD147 molecules [14].

As a ubiquitous monocarboxylate transporter, MCT1 is expressed in most human tissues, including erythrocytes and lymphocytes, where it mediates lactate efflux, and in the intestinal epithelium, cardiac myocytes, and red skeletal muscle fibres [15,16]. In cooperation with other monocarboxylate transporter isoforms, MCT1 plays an important role in metabolic regulation and the maintenance of tissue homeostasis.

While these canonical roles are well documented, recent studies have placed MCT1 in broader biological contexts, including immune cell function [17], tumour adaptation to metabolic stress [18,19,20], and possible links between monocarboxylate transport and chromatin regulation [17,21,22,23,24,25,26]. Importantly, these links should be interpreted at different levels of evidence. MCT1 has established functions in regulating the availability of metabolites such as lactate and pyruvate, which can influence epigenetic processes through independent chromatin-modifying mechanisms. By contrast, the possibility that MCT1 itself forms a functional nuclear pool with direct roles in chromatin regulation remains an emerging hypothesis requiring further experimental validation. This review therefore integrates established structural, metabolic, clinical, and pharmacological evidence with emerging non-canonical observations, while distinguishing validated mechanisms from testable models for future investigation.

## 2. Clinical Relevance of MCT1 in Cancer

The deregulation of cellular metabolism is now recognised as a core hallmark of cancer, enabling tumour cells to thrive in inhospitable microenvironments by rewiring energy production and nutrient use [29]. A prominent feature of this reprogramming is the glycolytic shift: tumours increase glucose uptake and convert pyruvate to lactate despite oxygen availability [30]. This state imposes a demand for efficient monocarboxylate transport, and tumours frequently upregulate PM MCT1 to sustain lactate and pyruvate flux across membranes. Although isoform contributions vary by tissue, immunohistochemistry and expression analyses consistently show altered PM MCT1 levels across malignancies, underscoring its role in metabolic adaptation (Table 1). Moreover, two independent studies have reported nuclear MCT1 staining in endometrial cancer and soft tissue sarcoma, where nMCT1 was associated with, or showed a trend towards, improved overall survival [31,32].

### 2.1. Clinical Relevance in Solid Tumours

In many solid tumours, overexpression of PM MCT1 is associated with aggressive disease and worse outcomes. Elevated PM MCT1 expression has been reported in non-small-cell lung carcinoma (NSCLC), cervical carcinoma, endometrial carcinoma, and glioblastoma, where it correlates with tumour progression and, in several cohorts, poor prognosis [34,46,47,48,49]. In pancreatic neuroendocrine tumours, a recent preprint reported heterogeneous MCT1 and MCT4 expression and proposed dual MCT1/MCT4 targeting as a strategy to address functional redundancy, although these findings require peer-reviewed validation [50]. In breast cancer, especially triple-negative disease, higher PM MCT1 levels link to shorter progression-free survival and a greater risk of recurrence [44]. Gynaecological cancers show a consistent pattern: in endometrial and ovarian adenocarcinomas, PM MCT1 overexpression predicts reduced overall survival and more advanced clinicopathological features [31,42]. Similar clinical associations appear across other epithelial tumours. In bladder cancer, patients with high PM MCT1 expression exhibit shorter overall survival and more frequent metastasis. Further, in oesophageal cancer, overexpression associates with lower overall and progression-free survival [40,45]. Head and neck cancers similarly show reduced overall and progression-free survival with high PM MCT1 levels [35]. Beyond carcinomas, mesenchymal tumours display congruent trends: in osteosarcoma, increased PM MCT1 correlates with poor prognosis, and pharmacological inhibition suppresses tumour growth in vitro and in vivo [41]. In soft tissue sarcomas, PM MCT1 associates with reduced overall survival, higher tumour grade, and disease progression [32]. In melanoma, PM MCT1 expression is more prevalent in metastatic lesions and links to shorter overall survival and adverse prognostic variables [36]. Collectively, these data indicate that, in most solid tumours, PM MCT1 expression marks metabolic plasticity that favours invasion, dissemination, and treatment resistance (Table 1).

### 2.2. Context-Dependent Exceptions

Not all tissues follow the same rule. Prostate cancer is a notable exception where PM MCT1 expression is reduced compared with normal tissue, suggesting distinct metabolic wiring and, possibly, a greater reliance on alternative monocarboxylate routes or isoforms [51]. Moreover, some NSCLC cohorts report high PM MCT1 expression as a positive prognostic marker, indicating that tumour context, tumour–stroma crosstalk, and isoform balance (e.g., MCT4) can invert clinical associations [39]. These exceptions do not diminish the clinical relevance of PM MCT1. Rather, they suggest that its prognostic value depends on tumour context. Across cancer types, PM MCT1 expression is most informative when interpreted alongside tumour subtype, metabolic phenotype, tumour microenvironmental features, subcellular localisation, and the expression of related transporters such as MCT4. This may help explain why PM MCT1 is associated with aggressive disease and poor outcome in many malignancies, while it has been linked to more favourable prognosis in selected NSCLC cohorts [39]. Similarly, the divergent prognostic implications of PM and nMCT1 in soft tissue sarcoma [32] and endometrial cancer [31] suggest that subcellular localisation may refine, rather than obscure, the biomarker value of MCT1. Together, these studies support MCT1 as a clinically relevant but context-dependent marker whose interpretation should account for isoform balance, metabolic phenotype, and tumour microenvironmental state.

### 2.3. Clinical Relevance in Haematological Malignancies

Across haematological malignancies such as B-cell and Burkitt’s lymphomas, non-Hodgkin lymphoma, and multiple myeloma, high PM MCT1 levels correlate with increased glycolytic activity, worse clinicopathological profiles, and inferior survival metrics [37,38,47,48,49]. In non-Hodgkin lymphoma, PM MCT1 overexpression tracks with poor progression-free and overall survival [38]. In multiple myeloma, PM MCT1 holds both prognostic and predictive value: overexpression is associated with reduced survival and impaired response to lenalidomide maintenance [37]. These findings reinforce the broader principle that elevated PM MCT1 often signals tumours that are metabolically adaptable and clinically more aggressive (Table 1). Taken together, these findings suggest that PM MCT1 overexpression is a common feature across diverse cancers, though its prognostic impact appears context-dependent. Together, these clinical studies establish MCT1 as a context-dependent biomarker across both solid and haematological malignancies. However, prognostic association alone does not establish therapeutic vulnerability. The following section therefore considers how MCT1 biology has been translated into pharmacological targeting, and why tumour metabolic context, particularly MCT4 expression and monocarboxylate dependency, is central to interpreting therapeutic potential.

### 2.4. From Biomarker to Therapeutic Target: Pharmacological Inhibition of MCT1

The breadth and consistency of these clinical observations have driven sustained efforts to therapeutically target MCT1. Early chemical probes—α-cyano-4-hydroxycinnamate (CHC) and analogues, stilbene disulfonates (DIDS, DBDS), phloretin, and bioflavonoids such as quercetin—were invaluable for defining transporter function but offered limited translational promise [7,52,53,54] due to specificity limitations. CHC inhibits mitochondrial pyruvate carriers roughly twice as potently as it inhibits MCT-mediated transport, while DIDS/DBDS also block the erythrocyte AE1 chloride/bicarbonate exchanger. Quercetin, on the other hand, affects multiple MCT isoforms [53,54,55]. These limitations sharpened the need for isoform-selective inhibitors with clean pharmacology (Table 2).

An unexpected clue from immunology accelerated this trajectory. Using photoaffinity labelling and proteomics, researchers identified MCT1 as the target of pyrrole–pyrimidine derivatives that selectively inhibit T-cell division and induce immunosuppression [63]. Compounds developed by AstraZeneca, AR-C117977, and AR-C122982 prevented acute allograft rejection in rats, mice, and non-vascularised transplant models, demonstrating in vivo potency against MCT1-dependent processes [64]. However, poor pharmacokinetics, including low oral bioavailability, limited their clinical prospects [65]. Medicinal chemistry optimisation yielded AR-C155858, a potent, selective MCT1 inhibitor that binds irreversibly from the cytosolic side to transmembrane helices 7–10 in a CD147-dependent manner [56]. Functionally, AR-C155858 blocks lactate export, acidifies the cytoplasm, lowers glycolytic rate, and suppresses tumour growth in Ras-transformed fibroblasts [66]. Importantly, hypoxia-driven MCT4 upregulation confers resistance, spotlighting a recurring theme: effective therapy may require dual targeting or combination strategies that address MCT4 compensation.

Building on these insights, AstraZeneca developed AZD3965, an orally bioavailable MCT1-selective inhibitor reported to have approximately six-fold selectivity for MCT1 over other MCT isoforms [58]. Preclinical studies demonstrated antitumour activity in small-cell lung cancer (SCLC) and haematological cancer models, including diffuse large B-cell lymphoma (DLBCL), non-Hodgkin lymphoma, and Burkitt lymphoma [58,59]. In SCLC models, MCT1 inhibition led to intracellular lactate accumulation and necrotic cell death, while efficacy also extended to lymphoma cell lines and the Raji xenograft model [58,59]. A Phase I clinical trial in patients with advanced solid tumours or lymphoma (NCT01791595, Cancer Research UK) reported a manageable safety and pharmacokinetic profile and included exploratory pharmacodynamic assessments such as FDG-PET and metabolomic readouts [60,61]. However, the trial also highlighted important translational barriers, including dose-limiting toxicities at higher dose levels, such as electroretinogram changes, increased cardiac troponin I, and acidosis [60,61]. A systematic review has since reinforced the preclinical antitumour activity of AZD3965 and noted enhanced efficacy in combination with other agents [67]. Together, these findings support continued investigation of AZD3965 in biomarker-selected contexts, particularly MCT1-high/MCT4-low tumours, while emphasising the need for toxicity monitoring, predictive biomarker validation, and strategies to address MCT4-mediated resistance. Mechanistically, MCT1 blockade can shift tumour cells toward mitochondrial metabolism, creating new liabilities. In lymphoma and colorectal carcinoma lines, MCT1 inhibition increased mitochondrial dependence; co-administration of metformin blunted this adaptation and enhanced cytotoxicity [68]. Combination therapy has therefore become a central theme. AZD3965 combined with doxorubicin, rituximab, or glutaminase (GLS) inhibitors outperformed monotherapy in lymphoma models, suggesting that glycolysis blockade plus OXPHOS interference or amino-acid metabolism targeting may be especially potent [69]. These insights have informed recommendations for future clinical trial designs that incorporate pre-planned combinations and metabolic biomarkers to guide patient selection and dosing [67].

Beyond AZD3965, additional chemotypes continue to probe MCT1 biology. 7-aminocarboxycoumarins (7ACC) selectively block lactate influx and show anticancer effects in mice, yet pharmacokinetic shortcomings and dosing challenges currently limit translation [57]. Syrosingopine, an antihypertensive with dual MCT1/MCT4 inhibitory activity, is synthetic-lethal with metformin, aligning with the metabolic liability concept; however, poor in vivo efficacy constrains its development and underscores the challenge of achieving balanced potency, selectivity, and exposure for dual-inhibition strategies [62,70]. Together, these programmes illustrate both the promise and the practical hurdles of targeting lactate transport in patients (Table 2).

### 2.5. Clinical Opportunities and Limitations of MCT1 Targeting

The therapeutic rationale for targeting MCT1 is strongest in tumours that depend on monocarboxylate transport and show high MCT1 expression with limited compensatory MCT4 activity. In preclinical lymphoma and small-cell lung cancer models, AZD3965 disrupted lactate transport, promoted intracellular lactate accumulation, and showed antitumour activity, supporting the development of MCT1-selective inhibitors [58,59]. MCT1 blockade can also create secondary metabolic vulnerabilities, including increased mitochondrial dependence, which may be exploited through rational combinations with agents such as metformin, chemotherapy, rituximab, or glutaminase inhibitors [68,69,71].

However, several barriers limit the use of MCT1 inhibitors as broadly effective monotherapies. First, MCT4 expression can reduce sensitivity to MCT1-selective inhibition by maintaining lactate transport capacity, particularly in hypoxic or metabolically plastic tumours [58,71]. Second, MCT1 has physiological roles in normal tissues, including the eye and heart, raising the possibility of on-target toxicity. In the Phase I evaluation of AZD3965, treatment-emergent adverse events were mostly low grade, but dose-limiting toxicities included ocular electroretinogram changes, cardiac troponin elevation, and acidosis at higher dose levels [60,61]. Third, predictive biomarkers for patient selection remain insufficiently validated. MCT1/MCT4 expression, FDG-PET changes, metabolomic readouts, and tumour metabolic phenotype may be useful as candidate biomarkers, but their clinical utility requires further prospective validation [60,61,67,68,69,71].

These limitations suggest that future development of MCT1 inhibitors will require biomarker-led patient selection, assessment of both MCT1 and MCT4 expression, and rational combinations designed to prevent metabolic escape. Rather than positioning MCT1 inhibition as a universal anticancer strategy, the strongest clinical opportunity may lie in identifying tumour contexts in which MCT1 represents a non-redundant metabolic dependency.

### 2.6. Clinical Outlook

To summarise, PM MCT1 expression is frequently associated with aggressive biology, adverse survival, and therapeutic resistance across solid tumours and haematological malignancies, establishing its value as a prognostic and, in contexts such as multiple myeloma, predictive biomarker [31,32,33,34,35,36,37,38,40,41,42,43,44,45,47,48,49]. Notably, prostate cancer and some NSCLC cohorts reveal context-dependence that likely reflects tumour-intrinsic metabolism, stromal interactions, hypoxia, and isoform balance (especially MCT4) [39,51]. On the therapeutic front, selective MCT1 inhibitors have advanced from tool compounds to clinical candidates, with AZD3965 providing the clearest path and combinations (e.g., with chemotherapy, rituximab, GLS inhibitors, metformin) offering a strategy to overcome metabolic rewiring and MCT4-mediated resistance [58,59,60,61,67,68,69]. The cumulative evidence positions MCT1 as both a clinically meaningful biomarker and a tractable drug target across multiple cancers.

Finally, the clinical picture is only part of the story. While canonical roles in metabolic transport explain much of MCT1’s prognostic and therapeutic relevance, emerging non-canonical functions—including nuclear localisation and possible epigenetic regulation—may further influence tumour behaviour and treatment response. Elucidating these roles, and integrating subcellular localisation with patient stratification, could refine how MCT1 is targeted and monitored in the clinic. The next section explores this evolving biology in detail.

## 3. Canonical Roles of MCT1 in Tumour Biology

### 3.1. Oncometabolites Beyond Lactate

**Figure 2:**
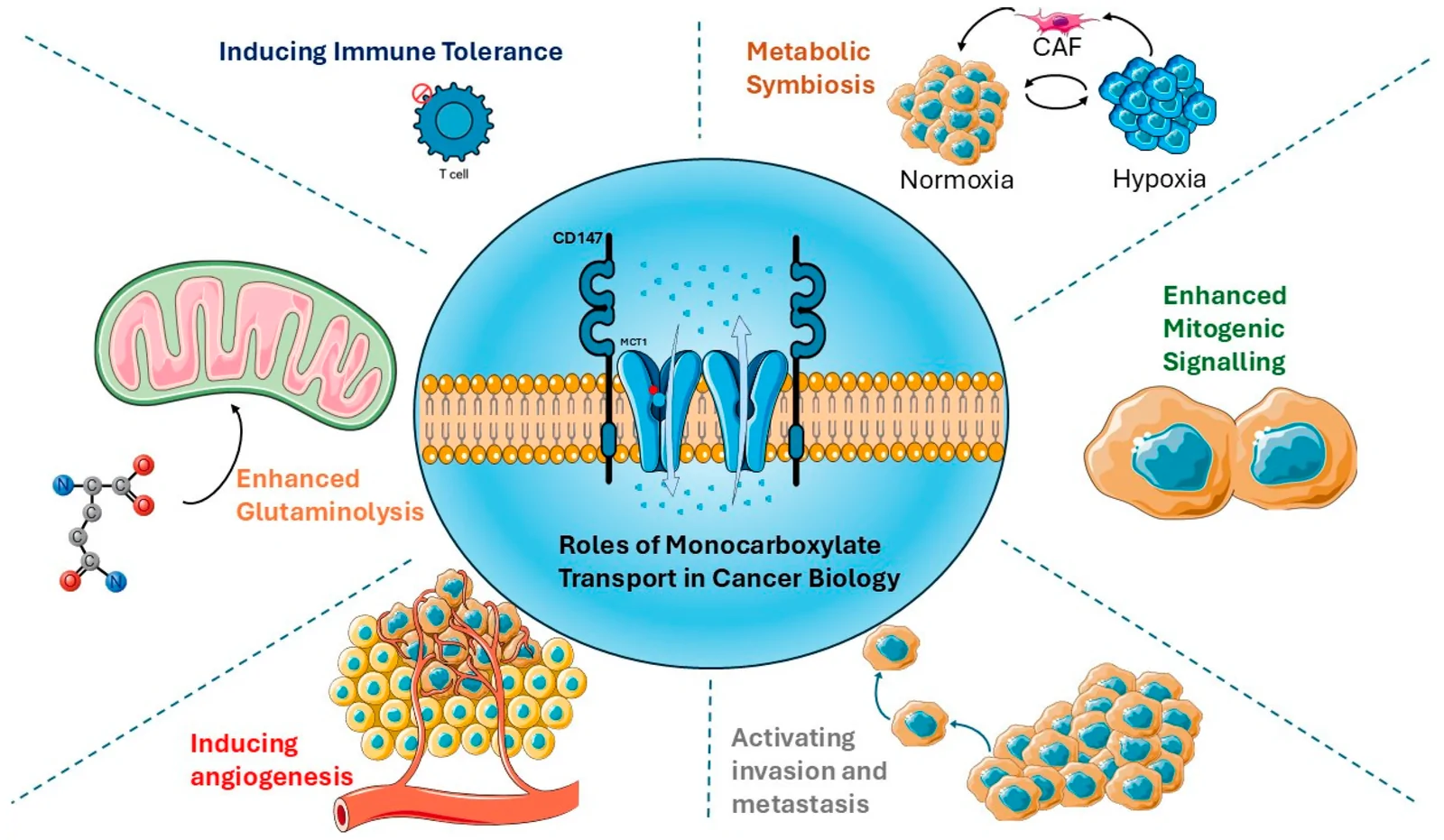
Canonical transport-dependent roles of PM MCT1 and related MCT isoforms in cancer. The figure summarises established transport-dependent roles of MCT1 and related MCT isoforms in cancer metabolic reprogramming. (1) MCT4-mediated lactate export from hypoxic cells and MCT1-mediated lactate uptake by oxygenated cells can facilitate metabolic cooperation between tumour cell populations. (2) Accumulation of oncometabolites within the cytoplasm and tumour microenvironment potentiates mitogenic signalling cascades, promoting tumour growth and proliferation. (3) PM MCT1 and its associated transport complex, including CD147, have been implicated in tumour cell growth, migration, and metastasis, in some contexts through mechanisms not fully explained by monocarboxylate transport alone. (4) PM MCT1-dependent lactate transport, and more broadly MCT-mediated monocarboxylate flux, can activate pro-angiogenic signalling pathways. (5) Chronic acidification of the tumour microenvironment as a result of MCT activity promotes a shift towards glutamine metabolism. (6) Monocarboxylate fluxes affect immune cell function through multiple immunomodulatory mechanisms. Arrows indicate the direction of metabolite transport or functional interactions between cellular compartments; symbols/icons represent the biological processes labelled in the figure; and colours are used to distinguish the different functional processes and cell/tissue compartments.

The role of PM MCT1 extends well beyond its connection to Warburg’s observation of accelerated glycolytic fluxes. Emerging evidence positions monocarboxylates as bona fide “oncometabolites” capable of shaping tumour behaviour [72,73]. Metabolic reprogramming frequently drives the accumulation of lactate, pyruvate, succinate, fumarate, and other intermediates in both intracellular and extracellular compartments, producing profound changes in tumour cell phenotypes through canonical MCT1-mediated transport (Figure 2). Although most reviews focus primarily on lactate, this is an incomplete view given the broader substrate specificity of PM MCT1 and select *SLC16A* transporters. For example, mutations or altered activities in lactate dehydrogenase, succinate dehydrogenase, and fumarate hydratase drive the overabundance of lactate, succinate, and fumarate, respectively [74]. These metabolites act as candidate biomarkers in multiple tumour types, including cancers of the uterus, breast, and prostate, as well as gliomas and endocrine malignancies [73,75]. Functionally, they regulate tumour aggressiveness and influence repopulation after therapy [76]. Transport of succinate by PM MCT1 has been reported in exercising muscle, ischaemic heart, and retinal epithelium [77,78,79]. However, succinate is considered a lower-affinity PM MCT1 substrate than canonical monocarboxylates such as lactate and pyruvate, and the relevance of MCT1-mediated succinate transport to tumour biology remains unclear. Clarifying the role of PM MCT1 in shuttling non-lactate oncometabolites may expand our understanding of oncogenesis and create new therapeutic opportunities.

### 3.2. Metabolic Symbiosis and the Reverse Warburg Effect

The best-characterised canonical function of MCT1 in tumours is its role in mediating metabolic symbiosis between heterogeneous cancer cell populations (Figure 2). Sonveaux and colleagues first demonstrated in 2008 that hypoxic tumour cells export lactate via MCT4, establishing a gradient that oxygenated cells exploit by importing lactate through MCT1 and feeding it into the TCA cycle [80]. This division of labour preserves glucose for hypoxic cells, supporting survival of the most glycolytic populations [81]. Disruption of this symbiosis using the MCT inhibitor CHC delayed tumour growth, radiosensitised oxygenated tumour rims, eradicated hypoxic niches, and expanded necrotic areas.

This work triggered extensive follow-up studies. A year later, Lisanti et al. described the “reverse Warburg effect,” in which tumour cells induce aerobic glycolysis in cancer-associated fibroblasts (CAFs), leading to secretion of lactate and pyruvate [82]. Mechanistically, tumour-derived ROS promote loss of caveolin-1 in CAFs, stabilising HIF1α and driving glycolysis. Curry et al. later showed that both processes—CAF-derived metabolite shuttling and intra-tumoural lactate symbiosis—can co-exist, particularly in head and neck cancer [83]. Here, MCT1 and MCT4 mediate the exchange of CAF-derived lactate and pyruvate between stromal and tumour compartments and serve as functional biomarkers of symbiotic metabolism. Evidence from additional tumour types links metabolic symbiosis to tumour aggressiveness, therapeutic resistance, and reduced patient survival [84,85,86].

### 3.3. Angiogenesis and Vascular Lactate Shuttling

Beyond tumour–tumour and tumour–stroma interactions, PM MCT1 also underpins communication between tumour cells and vascular endothelium. Lactate imported into endothelial cells activates HIF1α [87,88,89] and NF-κB, driving pro-angiogenic programmes through VEGFR2, IL-8, and basic fibroblast growth factor expression [90,91]. In parallel, signalling through the hydroxycarboxylic acid receptor HCAR1 amplifies angiogenic responses in breast cancer via CREB-mediated upregulation of amphiregulin [92]. Interestingly, not all MCT substrates promote angiogenesis. Butyrate, imported through PM MCT1, inhibits nuclear translocation of HIF1α in colon cancer cells, thereby suppressing angiogenesis, inhibiting proliferation, and promoting apoptosis [93]. Consistent with this, tumours arising from butyrate-rich tissues, such as colorectal cancers, often show reduced or silenced PM MCT1 expression [94]. These contrasting roles illustrate how the context of specific metabolites shapes PM MCT1’s impact on angiogenesis.

### 3.4. Crosstalk with Mitogenic Signalling Pathways

PM MCT1-mediated fluxes also intersect with key oncogenic signalling cascades. Intracellular lactate directly binds and stabilises NDRG3, preventing its proteasomal degradation. Accumulated NDRG3 activates c-RAF, thereby stimulating the MAPK/ERK pathway and driving mitogenic signalling [95]. Similarly, lactate accumulation inhibits the TSC2–Rheb complex, leading to activation of mTORC1 signalling independently of growth factor stimulation, as shown in *KRAS*-transformed MEF cells [96]. Lactate-driven extracellular acidosis in prostate carcinoma cells further enhances ROS generation, which, in turn, triggers ERK1/2 phosphorylation [97]. By contrast, uptake of β-hydroxybutyrate (BHB) by irradiated lung tumour cells suppresses mTOR activation, increases autophagy, and supports recovery after therapy [98]. These examples underscore how MCT1 activity integrates metabolic state with proliferative and survival signalling, in some contexts promoting tumour progression and in others mediating protective or adaptive responses.

### 3.5. Extracellular Matrix Remodelling

A further canonical role of PM MCT1 lies in regulating tumour cell invasion and metastasis, much of it mediated through its obligate chaperones. Both basigin (CD147) and embigin (GP70) are required for PM MCT1 trafficking and activity, but also possess independent signalling functions. As summarised by Grass et al., basigin forms homo-oligomers that stimulate matrix metalloproteinases (MMPs) through their N-terminal domains [99]. In breast cancer, CD147 upregulates and activates MMP14, promoting basement membrane degradation and enhanced cell motility [100]. In melanoma, co-culture of CD147-expressing tumour cells with dermal fibroblasts induces MMP1–3 production and facilitates invasion through basement membranes [101]. Because PM MCT1 depends on CD147 for functional expression, these pro-invasive and pro-angiogenic effects likely reflect the concerted action of the MCT1–CD147 complex. Coupled transport of lactate and pro-MMP signalling therefore represents a dual mechanism by which PM MCT1 reshapes the tumour microenvironment to support progression and dissemination. Increased MMP activity also enhances secretion of pro-angiogenic factors through multiple downstream pathways [102], further linking metabolic transport with structural remodelling of the tumour niche.

### 3.6. Metastasis and Tumour Progression

Beyond metabolic symbiosis, MCT1 directly contributes to tumour invasion and metastasis. In osteosarcoma and cervical carcinoma cells, MCT1 was shown to activate NF-κB signalling, thereby promoting migration and metastatic dissemination, although the precise molecular mechanism remains unresolved [103]. One proposed explanation is that MCT1 stabilises its chaperone CD147 at the plasma membrane, reducing internalisation or degradation of the MCT1–CD147 complex and prolonging CD147-driven downstream signalling. This sustained signalling could amplify pro-migratory and pro-inflammatory cascades such as NF-κB. Complementary studies in cervical carcinoma further reported that, under glucose starvation, MCT1 facilitated invasion in a manner dependent on mitochondrial ROS generation [104]. In line with this, breast cancer cells reabsorbing ketone bodies through MCT1 were found to promote lung metastasis in murine models, underscoring the transporter’s role in metabolic fuelling of metastatic spread [85].

### 3.7. Lactic Acidosis and Metabolic Remodelling

A hallmark of the tumour microenvironment (TME) is lactic acidosis, characterised by high extracellular lactate and reduced pH. Early profiling studies demonstrated that sustained lactic acidosis, driven by MCT1/4-mediated fluxes, promotes metabolic remodelling toward amino acid utilisation, particularly glutaminolysis, as a less productive proton-producing pathway that aids intracellular pH homeostasis [18]. Acidification of the extracellular milieu also restricts lactate/H+ efflux, causing NAD+ accumulation and activation of NAD+-dependent deacetylases such as SIRT1. This cascade promotes degradation of HIF1α, while stabilising HIF2α and inducing glutamine transporter (ASCT2) and glutaminase expression [19]. Follow-up studies in cervical carcinoma lines confirmed that intracellular lactate itself can stabilise HIF2α and activate c-Myc, shifting tumour metabolism toward glutaminolysis [20]. These findings emphasise that therapeutic inhibition of MCT1, or broader MCT-mediated lactate transport, cannot be considered in isolation from glutamine metabolism, given the extensive crosstalk between the two systems.

### 3.8. Immunosuppression and Tumour Immune Evasion

Perhaps the most profound systemic impact of MCT1 is on antitumour immunity. The initial identification of MCT inhibitors emerged from their ability to block T-cell activation in mice [63]. Activated lymphocytes rely on aerobic glycolysis for rapid proliferation and upregulate MCT1, in part due to NFAT-dependent promoter activity [105]. However, lactic acidosis in the TME disrupts continuous lactate efflux, thereby suppressing glycolytic fluxes and impairing T-cell activation, proliferation, and effector function [106,107]. Mechanistically, lactate selectively inhibits MAPK pathways downstream of the TCR, including JNK, c-Jun, and p38 in CD8+ lymphocytes [108]. Similar immunosuppressive effects extend to other immune subsets: dendritic cell migration, differentiation, and antigen presentation are suppressed under lactic acidosis, while HCAR1 signalling in tumour cells enhances PD-L1 expression through YAP/TAZ activation and simultaneously reduces MHC class II expression in dendritic cells [109,110,111].

The immunomodulatory reach of monocarboxylates is not restricted to lymphocytes. In breast cancer, lactic acidosis skews macrophage differentiation from the pro-inflammatory M1 to the tumour-supportive M2 phenotype [111]. Succinate secreted by lung tumour cells into the TME similarly promotes M2 polarisation, raising the possibility that monocarboxylate transporters, including MCT1 in tissues where succinate transport has been reported, may contribute to this process [77,112]. In contrast to lactate and succinate, ketone bodies can confer immune-stimulatory effects: supplementation with 3-hydroxybutyrate was reported to suppress PD-L1 expression on tumour cells in murine models [113]. Butyrate likewise activates multiple immune subsets, including T and B lymphocytes and macrophages [114]. Notably, despite these opposing effects, MCT1 inhibition did not synergise with 3-hydroxybutyrate supplementation in prostate cancer xenografts, highlighting the complexity of targeting monocarboxylate fluxes in immunometabolism [115].

Taken together, the canonical functions of MCT1 extend well beyond simple lactate shuttling. By fuelling metastasis, reshaping metabolism under lactic acidosis, and reprogramming immune responses, MCT1 emerges as a central regulator of the tumour ecosystem. Notably, monocarboxylate substrates themselves can directly modify proteins through post-translational modifications, influencing immune cell phenotypes and highlighting the emerging field of immunometabolism [116]. These findings demonstrate how MCT1-dependent and broader MCT-mediated monocarboxylate fluxes can integrate tumour metabolism with immune evasion and provide mechanistic links between metabolic adaptation, tumour progression, and immune suppression. Understanding this complexity is essential not only for appreciating the breadth of MCT1’s canonical functions but also for contextualising the emerging evidence of its non-canonical roles in nuclear localisation and epigenetic regulation, which are explored in the following section.

## 4. Emerging Non-Canonical Functions and the Unique Role of Subcellular Localisation of MCT1

It is increasingly recognised that changes in the tumour transcriptome are not dictated solely by mutations in oncogenes and tumour suppressors. Instead, metabolic reprogramming itself can drive heritable, epigenetic alterations that shape gene expression without altering the DNA sequence [21]. Epigenetic regulation encompasses chromatin reconfiguration, non-coding RNAs, and post-translational modifications (PTMs) of histones, which, together, enable tumour cells to rapidly adapt to environmental stresses. Among these, metabolite-driven histone modifications have emerged as key links between metabolism and gene expression, with availability of metabolites such as acetyl-CoA, α-KG, and lactate serving as direct substrates or cofactors for chromatin-modifying enzymes [21]. In this section, we distinguish three related but mechanistically distinct concepts. First, PM MCT1 can regulate the availability of monocarboxylates, including lactate, pyruvate, and ketone bodies, that are known to influence chromatin-regulatory processes. Second, PM MCT1 inhibition or genetic loss may alter histone modifications indirectly by changing cellular metabolism and substrate availability. Third, a putative nuclear or nuclear-associated pool of MCT1 has been reported in selected contexts, but its direct function remains unresolved.

### 4.1. Metabolic Moonlighting and Nuclear Compartmentalisation

Eukaryotic cells regulate the localisation of metabolites across subcellular compartments to control diverse biological processes. In tumour cells, however, metabolic reprogramming leads to unusual shifts in metabolite pools within specific compartments, particularly the nucleus. These shifts are increasingly thought to be facilitated by the secondary “moonlighting” functions of proteins that become overexpressed in response to oncogenic or metabolic stress [117]. Moonlighting proteins are capable of performing multiple distinct functions using a single polypeptide chain, independent of gene fusions, alternative splicing, or pleiotropy [118].

Moonlighting is especially common among proteins involved in metabolic regulation, including enzymes, GPCRs, ion channels, chaperones, and membrane transporters [119]. Due to their evolutionary conservation, these proteins may have been under selective pressure to develop secondary functions that enhance adaptability. As reviewed by Boukouris et al., metabolic moonlighting frequently occurs through relocalisation of proteins to alternative subcellular compartments, most often the nucleus [120]. This nuclear localisation economises protein function by directly linking cellular metabolic state with transcriptional regulation.

Moonlighting proteins can influence chromatin either indirectly, by generating substrates for chromatin modifiers, or directly, by shaping the metabolic microenvironment at chromatin-associated domains. Recent studies highlight that chromatin may act as a genome-wide reservoir for metabolites, with changes in nuclear metabolite abundance tightly linked to gene transcription [121]. An emerging paradigm is that moonlighting proteins localise to phase-separated nuclear microdomains where they interact with chromatin modifiers, either by altering their enzymatic activity or by locally supplying metabolites required for PTMs [122,123]. MCT1 has not yet been demonstrated to fulfil the strict criteria of a moonlighting protein. Direct evidence for a nuclear-specific binding partner, distinct nuclear conformation, chromatin-associated activity, or DNA/RNA-binding function remains lacking. Nevertheless, several observations make this framework relevant for future study, including reports of nuclear or nuclear-associated MCT1 staining, co-localisation with CD147 at the nuclear membrane, and evidence that MCT1-dependent metabolite availability can influence histone acetylation and transcriptional regulation in specific cellular contexts [17,31,32,124,125].

### 4.2. Evidence for Nuclear Localisation of MCT1

**Figure 3:**
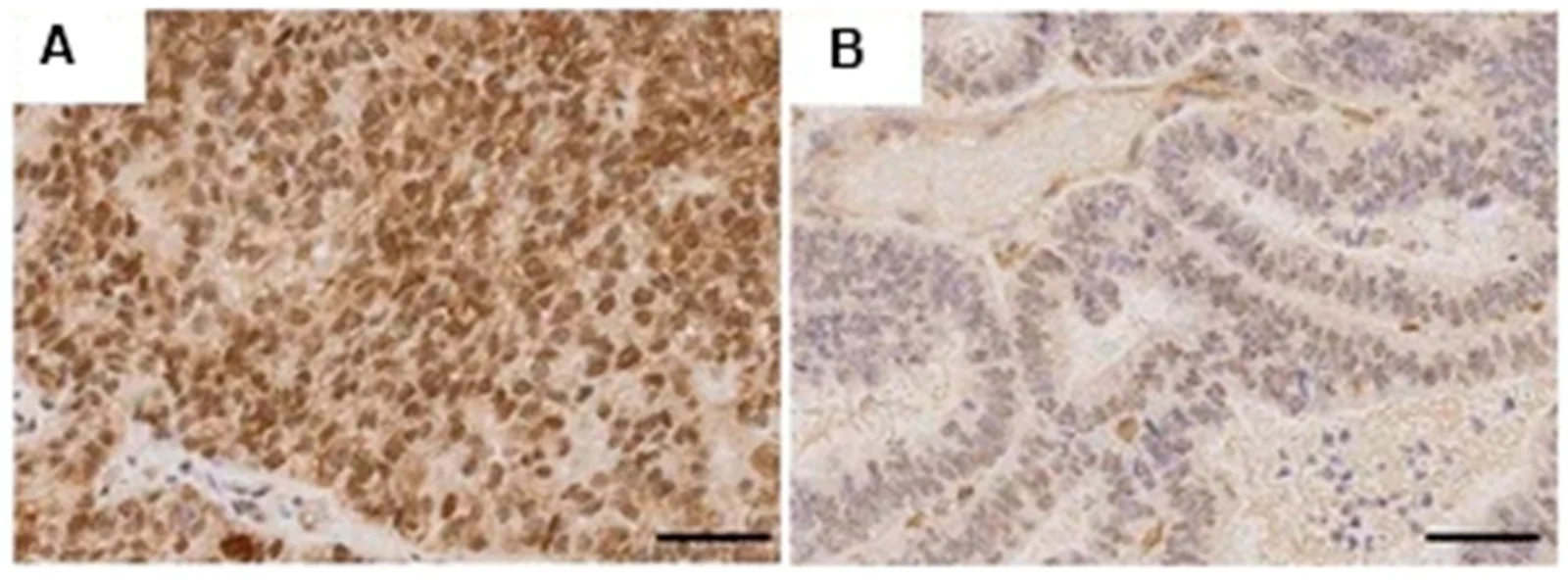
Published immunohistochemical evidence of nuclear MCT1 staining in endometrial cancer tissue [31]. (**A**,**B**) Representative immunohistochemistry images adapted from Latif et al. showing MCT1 staining in endometrial cancer tissue sections. Panels show tumour regions in which nuclear MCT1 immunoreactivity was reported in malignant epithelial cells. These images illustrate previously published observations of nuclear MCT1 staining by immunohistochemistry; scale bars represent 50 µm.

Nuclear localisation of transporters is unusual, but several members of the SLC superfamily, including glucose transporters and monocarboxylate transporters, have been reported in the nucleus using immunohistochemistry (IHC) and immunofluorescence (IF) (Figure 3). These reports raise the possibility that MCT1 may occupy nuclear or nuclear-associated compartments in selected contexts. nMCT1 was first reported in human granulocytes, lymphocytes, and monocytes, with IHC demonstrating its presence at the nuclear membrane in all three immune cell types [123].

Subsequent studies extended these observations to tumour contexts. Although Valença et al. primarily focused on MCT2 localisation at peroxisomes in prostate cancer, their study also reported MCT1 nuclear localisation as a secondary observation, with strong nuclear MCT1 staining in PNT1A, 22Rv1, and PC3 prostate-derived cell lines and CD147 co-localising at the nuclear envelope in 22Rv1 cells (page 726, Figure 2A) [124]. This supports nuclear or nuclear-associated MCT1 staining in selected prostate-derived epithelial models, although the functional significance of this localisation remains unresolved. Similarly, nMCT1 staining has been reported in more than 50% of endometrial cancer samples and in soft tissue sarcomas, where it correlated with more favourable prognostic features and lipogenic cell lineage identity [31,32]. The studies reporting nuclear localisation of the transporter are summarised in Table 3.

Although nuclear MCT1 staining has been reported by immunohistochemistry and immunofluorescence, imaging alone cannot definitively establish a functional nuclear pool of MCT1. Such observations may be influenced by antibody specificity, fixation conditions, optical resolution, and overlap between nuclear, perinuclear, and membrane-associated signals. Therefore, nuclear MCT1 staining should be interpreted cautiously and validated using orthogonal approaches such as subcellular fractionation, Western blotting, or targeted proteomics and MCT1-deficient controls.

### 4.3. Functional Implications in Immune Cells

The strongest mechanistic evidence linking MCT1 activity to metabolic–epigenetic regulation comes from activated murine B cells. In vivo murine studies revealed that MCT1 is essential for antibody class-switch recombination (CSR) [17]. Using a genetic knockout model, investigators showed that MCT1-dependent pyruvate transport promotes expression of activation-induced cytidine deaminase (AID), the enzyme initiating CSR. MCT1-deficient B cells displayed reduced AID expression, accompanied by global reductions in histone acetylation (H3K9, H3K14, H3K27). Genome-wide profiling with ATAC-Seq and ChIP-Seq revealed significantly fewer acetylated promoter regions in MCT1-deficient cells. Collectively, these findings suggest that MCT1 modulates nuclear metabolite availability to regulate histone acetylation and transcriptional programmes essential for adaptive immunity. These findings provide important evidence that MCT1-dependent metabolite availability can influence histone acetylation and transcriptional programmes. However, this study did not directly demonstrate nuclear localisation of MCT1, and its relevance to putative nuclear MCT1 function in cancer remains to be tested. Nevertheless, it provides a mechanistic rationale for investigating whether MCT1-mediated monocarboxylate transport can shape chromatin regulation in tumour and immune contexts.

### 4.4. Links to Lipid Metabolism

MCT1 localisation has also been linked to metabolic pathways supporting lipogenesis. Import of CAF-derived lactate via MCT1 was previously shown to drive lipid droplet accumulation in prostate cancer through activation of lipogenic transcription factors [125]. These droplets act as a source of acetyl groups, fuelling histone acetylation and tumour progression. Nuclear localisation of MCT1 in soft tissue sarcomas is correlated with lipogenic lineage identity, raising the possibility that putative nuclear MCT1 could be associated with chromatin–lipid metabolism feedback loops, although this remains speculative [32]. Although direct evidence remains lacking, a plausible hypothesis is that nMCT1 regulates metabolite availability—such as lactate or pyruvate—to support histone acetylation at lipogenic gene promoters. Such compartmentalised metabolite channelling within nuclear microdomains could enhance the efficiency of metabolic–epigenetic crosstalk, a concept ripe for experimental exploration.

### 4.5. Mechanisms of Nuclear Trafficking

Validation of nuclear or nuclear-associated MCT1 would imply dedicated trafficking or retention mechanisms. In epithelial cells, plasma membrane localisation of MCT1 is independent of classical sorting motifs in its cytoplasmic tails and relies entirely on its association with CD147 [13]. This is unexpected given that MCT1 contains several protein–protein interaction and trafficking motifs in its intracellular domains (Figure 1). How these motifs contribute to nuclear trafficking remains unknown. At present, neither the molecular signals directing nuclear import nor the vesicular pathways responsible have been characterised [128]. Clarifying these mechanisms will be critical to understanding whether nuclear trafficking is selectively activated in specific contexts, such as metabolic stress or oncogenic signalling.

### 4.6. Epigenetic Regulation via MCT1 Substrates

Independent of nuclear trafficking, several MCT1 substrates can act as chromatin regulators. Extracellular lactate can inhibit histone deacetylases (HDACs), causing histone hyperacetylation, reduced chromatin compaction, and enhanced DNA-PK activity at tumour cell nuclei [22,129]. Ketone bodies such as β-hydroxybutyrate (BHB) similarly inhibit class I HDACs [23], while accumulation of α-ketoglutarate (α-KG) in PDAC stroma activates TET demethylases, leading to histone hydroxymethylation [24].

Recently, novel histone PTMs directly linked to MCT1 substrates have been identified. Lactate acts as a substrate for histone lactylation, mediated by p300 HAT, functioning as a transcriptional “timer” for wound-healing genes in immune cells [25]. Histone lactylation has also been observed in NSCLC, where it drives metabolic reprogramming through lactylation-mediated gene expression [26]. Likewise, β-hydroxybutyrylation represents a new layer of epigenetic regulation tied to ketone body metabolism.

### 4.7. Tumoural and Immune Crosstalk

In prostate cancer cells, lactate import through MCT1 has been reported to stabilise HIF1α under normoxic conditions via HIF1α lactylation, promoting transcription of HIF1α target genes [126]. In gliomas, hypoxia-induced lactate accumulation has been reported to promote macrophage M2 polarisation through lactate uptake in macrophages and activation of an MCT1/H3K18La/TNFSF9 axis [127]. These findings support a role for MCT1-mediated metabolite flux in tumour and immune-cell signalling, but do not establish a direct nuclear function for MCT1. Such mechanisms highlight the growing field of immunometabolism, where metabolite-mediated and MCT-associated pathways may reshape tumour and immune cell behaviour, while the contribution of nuclear or nuclear-associated MCT1 remains to be defined.

## 5. Conclusions

MCT1 is a clinically relevant regulator of cancer metabolism with established roles in monocarboxylate transport, metabolic symbiosis, tumour adaptation, immune modulation, and therapy resistance. Its expression has prognostic or predictive value in several malignancies, although these associations remain context-dependent and are influenced by tumour type, subcellular localisation, metabolic phenotype, and the balance between MCT1 and other transporters such as MCT4. Therapeutic targeting of MCT1, particularly through selective inhibitors such as AZD3965, remains a promising strategy, but clinical translation will require improved patient stratification, management of compensatory resistance mechanisms, and rational combination approaches.

Beyond its canonical transport functions, MCT1 may also contribute to metabolic–epigenetic crosstalk by regulating the availability of metabolites such as lactate, pyruvate, and ketone bodies, which can influence histone modifications and transcriptional programmes through established chromatin-regulatory mechanisms. Reports of nuclear or nuclear-associated MCT1 staining in immune and cancer contexts raise the possibility of additional non-canonical roles. However, direct nuclear functions of MCT1 remain insufficiently validated, and current models should be considered hypotheses requiring orthogonal experimental confirmation.

Future work should therefore prioritise rigorous validation of putative nuclear MCT1 localisation and function using complementary biochemical, imaging, proteomic, and genetic approaches. Distinguishing direct nuclear roles from indirect consequences of altered substrate transport will be essential for defining whether nuclear or nuclear-associated MCT1 represents a functional regulator of chromatin biology, a biomarker of tumour metabolic state, or both. A more precise understanding of these mechanisms will help determine how MCT1 biology can be exploited for cancer prognosis, patient stratification, and therapy.

## Figures and Tables

**Table 1:** Clinical significance of MCT1 expression and subcellular localisation across cancer types. PM MCT1: plasma membrane MCT1; nMCT1: nuclear MCT1.

Tumour Type	Clinical Significance	Clinical Features
Clear cell renal cell carcinoma [33]	Prognostic negative (PM MCT1)	Reduced overall survival
Endometrial Cancer [31]	Prognostic negative (PM MCT1) Prognostic positive (nMCT1)	PM MCT1—reduced overall survival nMCT1—trend towards association with improved survival for nMCT1.
Glioblastoma [34]	Prognostic negative (PM MCT1)	Reduced overall survival
Head and neck squamous cell carcinoma [35]	Prognostic negative (PM MCT1)	Reduced overall and progression-free survival
Melanoma [36]	Prognostic negative (PM MCT1)	Reduced overall survival, poor prognostic variables
Multiple myeloma [37]	Prognostic negative, predictive (PM MCT1)	Reduced overall and progression-free survival, predictive for lenalidomide maintenance therapy
Non-Hodgkin Lymphomas [38]	Prognostic negative (PM MCT1)	Reduced overall survival, poor clinicopathological profile
Non-small-cell lung cancer (NSCLC) [39]	Prognostic positive (PM MCT1)	Increased progression-free survival
Oesophageal squamous cell carcinoma [40]	Prognostic negative (PM MCT1)	Reduced overall survival and progression-free survival
Osteosarcoma [41]	Prognostic negative (PM MCT1)	Decreased overall survival
Ovarian adenocarcinoma [42]	Prognostic negative (PM MCT1)	Poor clinicopathological profile
Soft tissue Sarcoma [32]	Prognostic negative (PM MCT1) Prognostic positive (nMCT1)	PM MCT1—decreased overall survival nMCT1—improved overall survival
Testicular germ cell cancer [43]	Prognostic negative (PM MCT1)	Poor clinicopathological profile
Triple-negative Breast Cancer [44]	Prognostic negative (PM MCT1)	Reduced progression-free survival, increased recurrence risk
Urothelial cancer [45]	Prognostic negative (PM MCT1)	Reduced overall survival, Increased metastasis risk

**Table 2:** Selected inhibitors and tool compounds targeting MCT1 or related monocarboxylate transport pathways in cancer.

Inhibitor Name	Mechanism of Action	Development Stage/Status	Relevance in Cancer Therapy	Key Findings	Limitations
CHC [7,55]	Non-specific MCT1/MCT2 and pyruvate carrier inhibitor	Research tool; non-selective inhibitor	Early tool compound to study lactate transport in cancer metabolism	Blocks lactate export; reduces tumour cell proliferation in vitro	Non-selective; inhibits other transporters (e.g., pyruvate carriers)
DIDS [52]	Broad-spectrum anion transporter inhibitor	Research tool; broad-spectrum anion transporter inhibitor	Used to study lactate shuttle disruption in hypoxic tumours	Reduces lactate efflux; disrupts tumour–stroma metabolic coupling	Highly non-specific (affects bicarbonate exchangers, chloride channels); toxic
DBDS [52]	Broad-spectrum MCT inhibitor	Research tool; broad-spectrum MCT inhibitor	Investigated for targeting glycolytic tumours	Inhibits lactate transport; synergises with glycolysis inhibition in vitro	Limited in vivo data; unclear pharmacokinetics
Quercetin [54]	Non-specific MCT1/MCT2 inhibitor	Research tool; non-selective natural product	Tested as adjuvant therapy in vitro	Enhances chemosensitivity; reduces tumour lactate in combination models	Low potency; non-specific for MCT1
AR-C155858 [56]	Dual MCT1/MCT2 inhibitor	Preclinical	Disrupts lactate shuttle in hypoxic tumours	Reduces tumour growth in xenografts; increases oxidative stress	Off-target MCT2 inhibition; toxicity concerns limit clinical translation
7ACC2 [57]	Selective MCT1 inhibitor	Preclinical	Targets lactate-dependent tumours	Inhibits lactate influx; shows antitumour activity in mouse models	Poor pharmacokinetics; limited in vivo data
AZD3965 [58,59,60,61]	Selective MCT1 inhibitor	Clinical, phase I dose escalation/expansion	Targets tumours dependent on MCT1-mediated lactate transport, particularly MCT1-high/MCT4-low contexts	Demonstrated target engagement and pharmacodynamic activity; preclinical antitumour activity in lymphoma and SCLC models	MCT4 expression may confer resistance; dose-limiting toxicities included ocular electroretinogram changes, cardiac troponin elevation and acidosis at higher dose levels; predictive biomarkers remain unvalidated
Syrosingopine [62]	Dual MCT1/MCT4 inhibitor	Preclinical	Targets highly glycolytic tumours	Blocks lactate efflux; induces acidification and apoptosis	Limited selectivity; potential cardiovascular side effects (historical use as antihypertensive)

**Table 3:** Summary of evidence for nuclear localisation of MCT1.

Study	Cell Type/Tissue	Method Used	Key Findings	Additional Notes
Merezhinskaya et al. [123]	Human leukocytes (granulocytes, monocytes, lymphocytes)	Immunohistochemistry (IHC)	Nuclear membrane-associated MCT1 staining reported	First report of nuclear MCT1; suggests immune-specific regulatory functions
Chi et al. [17]	Murine B cells (genetic KO model)	Genetic knockout, ChIP-Seq, ATAC-Seq	MCT1-dependent pyruvate metabolism regulates histone acetylation and CSR	Supports a functional link between MCT1 activity and chromatin regulation; nuclear localisation of MCT1 was not directly assessed
Pinheiro et al. [32]	Soft tissue sarcomas (myxoid liposarcoma)	Immunofluorescence (IF)	Nuclear MCT1 staining associated with better prognosis and lipogenic lineage.	Points to a metabolic–epigenetic link and prognostic significance
Valença et al. [124]	Prostate cancer cell lines	Immunofluorescence (IF)	MCT1 nuclear localisation reported on page 726 and in Figure 2A of the original Valença et al. study [124].CD147 co-localised at the nuclear envelope in 22Rv1 cells	Supports nuclear or nuclear-envelope-associated MCT1 staining in selected prostate-derived epithelial models. Functional significance remains unresolved
Latif et al. [31]	Endometrial Cancer (FFPE tissue)	Immunohistochemistry (IHC)	Nuclear MCT1 staining reported in >50% of tumour samples	Potential biomarker relevance
Additional studies [17,22,23,24,25,26,126,127]	Various cancer models and immune cells	Biochemical and in vitro assays	Indirect evidence via substrates (lactate, BHB) influencing histone PTMs	MCT1-transported metabolites (lactate, BHB) shown to modulate chromatin structure and gene expression

## Data Availability

No new datasets were generated or analysed in this review. Data supporting the discussion are available from the cited literature. Representative images shown in Figure 3 are available from the corresponding author upon reasonable request.
